# The Interference Mechanism and Regularity Analysis of Gas Pipelines Affected by High-Speed Rail Based on Field Testing and Numerical Simulation

**DOI:** 10.3390/ma18174203

**Published:** 2025-09-08

**Authors:** Yuxing Zhang, Caigang Ge, Ziru Chang, Yanxia Du, Minxu Lu, Zitao Jiang

**Affiliations:** 1 Corrosion and Protection Center, Institute of Advanced Materials and Technology, University of Science and Technology Beijing, Beijing 100083, Chinaduyanxia@ustb.edu.cn (Y.D.);; 2 Research Institute, Beijing Gas Group Co., Ltd., Beijing 100035, China; 3 Beijing Cathtop Technology Co., Ltd., Beijing 100020, China; 4 National Engineering Laboratory for Pipeline Safety/Beijing Key Laboratory of Urban Oil and Gas Distribution Technology, China University of Petroleum-Beijing, Beijing 102249, China

**Keywords:** interference patterns, high-speed railway, gas pipeline, electromagnetic induction coupling interference, interference mechanism, interference patterns

## Abstract

By monitoring the alternating interference voltage at the intersections and parallels of gas pipelines with high-speed railways, the alternating voltage between the high-speed railway track supports and the ground, the alternating ground voltage gradient along parallel and perpendicular high-speed railway tracks, and the timing of train passages, the interference patterns caused by high-speed railways on pipelines are analyzed. A numerical model was developed to elucidate interference mechanisms. The conclusions indicate that the interference caused by the parallel and intersecting presence of high-speed railways and pipelines is far greater than that caused solely by the intersection of railways and pipelines. The peak alternating voltage interference on pipelines occurs at the insulation joints of the pipelines, the positions of the pipelines corresponding to the high-speed railway track circuits (AT), and the positions of the pipelines corresponding to the passage of trains. The alternating interference caused by high-speed railway lines on pipelines involves both resistive coupling interference and electromagnetic induction coupling interference, with the latter dominating.

## 1. Introduction

With the rapid development of energy and transportation industries, a complex network of energy transmission and transportation has been formed, which includes high-speed electrified railways and buried oil and gas pipelines that are widely distributed across the country. High-speed rail is an important infrastructure for national transportation and plays a significant role in the overall transportation system. As of the end of 2024, the total operating mileage of railways in China has reached 162,000 km, including 48,000 km of high-speed railways. Meanwhile, to meet the increasing energy demand in various regions, China has added 932,000 km of urban natural gas pipelines from 2006 to 2020, with 194,000 km added during the 11th Five-Year Plan period, 306,000 km added during the 12th Five-Year Plan period, and 432,000 km added during the 13th Five-Year Plan period. As of 2024, the scale of China’s urban natural gas pipeline network has reached 1,206,000 km. Due to limited geographical resources, situations where high-speed rail and natural gas pipelines run parallel or intersect cannot be avoided. Buried steel pipelines will be affected by the alternating current interference from high-speed rail, leading to an increased risk of pipeline corrosion [1,2].

In recent years, scholars have recognized the corrosion risks that electrified railways pose to pipelines and have gradually initiated research into monitoring, detection, and interference mechanisms. Guo Qingru et al. [3] conducted a study on the oil pipeline network adjacent to the Harbin–Dalian Railway, revealing that the majority of pipeline segments experienced interference voltages exceeding 10 V. In 2006, a certain inspection company monitored a section of finished oil pipeline, which runs parallel to the Guiyang–Kunming Railway and accounts for 40% of its length. By measuring the alternating current voltage values on the railway tracks when trains passed through and the pipeline’s alternating current voltage values, it was discovered that the pipeline’s voltage fluctuations were positively correlated with those of the electrified railway tracks [4]. Furthermore, various entities in cities such as Urumqi, Hunchun, and Zhenjiang have successively discovered dynamic AC interference from electrified railways on nearby pipelines, with the maximum interference current density reaching 393 A/m^2^ [5,6,7,8,9]. Similar occurrences of AC corrosion have also been observed in buried pipelines adjacent to AC electrified railways in countries such as Austria and Canada [10,11,12,13].

In this study, we selected natural gas pipelines that intersect parallel to high-speed railways for alternating current interference detection. By testing the AC voltage distribution along the pipeline, the AC potential gradient between the parallel and perpendicular positions relative to the high-speed railway, and the AC voltage induced by the high-speed railway’s support piers, we analyzed the patterns of AC interference exerted by the high-speed railway on the pipeline and identified the influencing factors. Furthermore, we sought to determine whether the dominant form of interference from the high-speed railway is resistive or inductive coupling. This research aims to provide technical support for the assessment of AC interference risks and the selection of protective measures. The interference mechanisms and findings of this work are based on fundamental electromagnetic principles and pipeline properties and are therefore generally applicable to similar high-speed railway and pipeline configurations in other regions worldwide.

## 2. Discussion on the Forms of AC Interference on Pipelines from High-Speed Railways

The forms of AC interference on in-service buried pipelines caused by high-speed railways mainly consist of resistive coupling and inductive coupling interference. Resistive coupling is a result of stray currents generated when traction current flows through the steel rails and dissipates into the ground during train operation. Inductive coupling arises from the alternating magnetic field induced by the AC current, leading to the generation of induced currents in the adjacent pipeline, causing interference.

Zhu Jiuguo [14] proposed that electrified railway power supply systems have both inductive and resistive coupling effects on buried pipelines. He derived mathematical models for calculating the interference voltage of different coupling modes through numerical simulations. However, the research did not specifically investigate which coupling mode dominates the extent of pipeline interference. C. A. Charalambous et al. [15] suggested that AC electrification systems have two main sources of interference: overhead contact lines and traction circuits (i.e., the tracks). While attention is given to induced voltage, the importance of resistive coupling voltage should not be overlooked. Zhang Xiaoyue et al. [16] and Li Xingfeng et al. [17] proposed that there is not complete insulation between the track and the ground, and therefore, the traction power supply system primarily causes interference to adjacent buried pipelines through resistive coupling. Andrea Mariscotti [18], Václav Kolář [19], and others have clarified the proportion of return current to leakage current through injecting current signals, and field tests have demonstrated that a portion of the return current from the traction system leaks into the ground through the rails, resulting in interference on the buried pipelines. Sun Peiqi et al. [20] and Braunstein et al. [21] proposed that inductive coupling is the predominant form of interference on pipelines parallel to the track.

Based on the discussions above, it is evident that there are different viewpoints regarding the main forms of interference caused by high-speed railways. and the dominant role of resistive coupling versus inductive coupling remains controversial. On this basis, researchers have further expanded the scope of investigation to focus on key factors affecting the intensity of AC interference. These factors mainly include traction load parameters [22], pipeline parameters [23], and soil structure parameters [24].

In summary, multiple perspectives still exist concerning both the forms and influencing factors of pipeline interference caused by high-speed railways. The clarification of interference forms is crucial for determining numerical simulation models, defining boundaries, assessing interference risks, and selecting appropriate protective measures. Therefore, systematic research on interference forms is urgently needed.

## 3. Measurement Methods

To evaluate the AC interference, several types of measurements were conducted.

**Figure 1:**
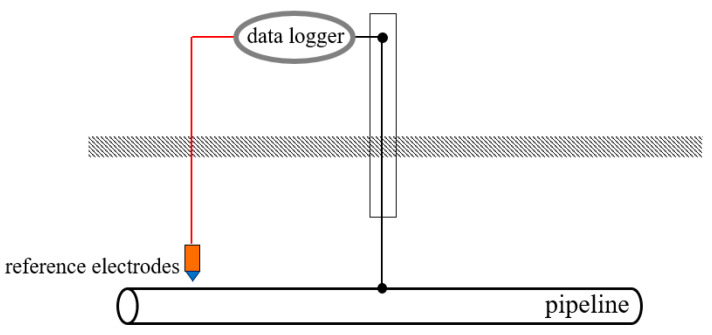
Wiring diagram for AC voltage measurement.

First, data loggers together with copper sulfate reference electrodes were positioned at designated locations along the pipeline to record the AC voltage. The AC voltage at each testing point was synchronously measured, with the data acquisition frequency set to one set per second. The wiring configuration for the measurement is shown in Figure 1.

**Figure 2:**
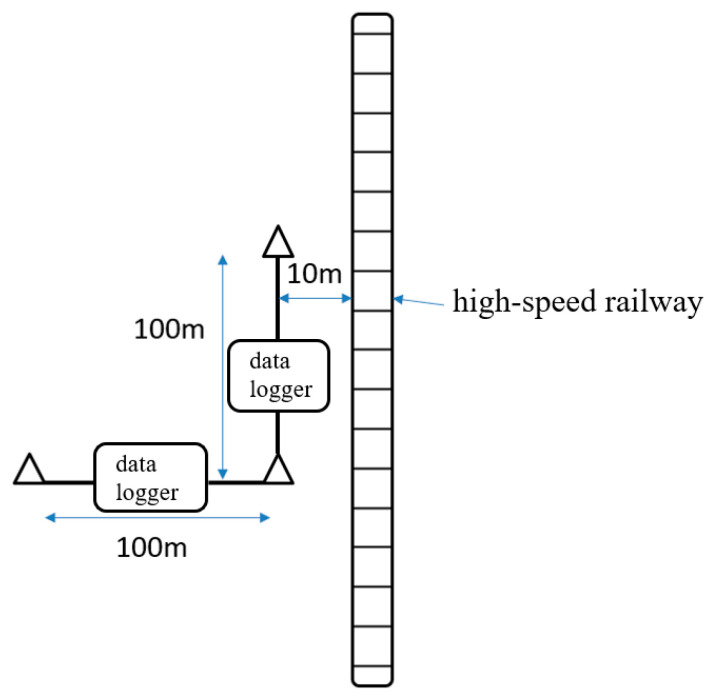
Illustration of AC ground potential gradient measurement.

Second, the AC ground potential gradient was measured at a distance of 10 m from the high-speed railway using four reference electrodes and a data logger. The acquisition frequency was also set to one set per second. Additional measurements were performed at 100 m parallel and vertical to the railway, as shown in Figure 2.

Third, the AC voltage between the pier of the elevated high-speed railway bridge and the surrounding ground was monitored. The potential difference was measured between a reference electrode placed 0.5 m from the pier and another placed 30 m away, using a data logger.

Finally, during the measurements of pipeline AC voltage, AC ground potential gradient, and pier-to-ground AC voltage, manually record the specific timing of train passages at the testing points.

## 4. Basic Overview of Three Pipelines and High-Speed Railway Line

**Figure 3:**
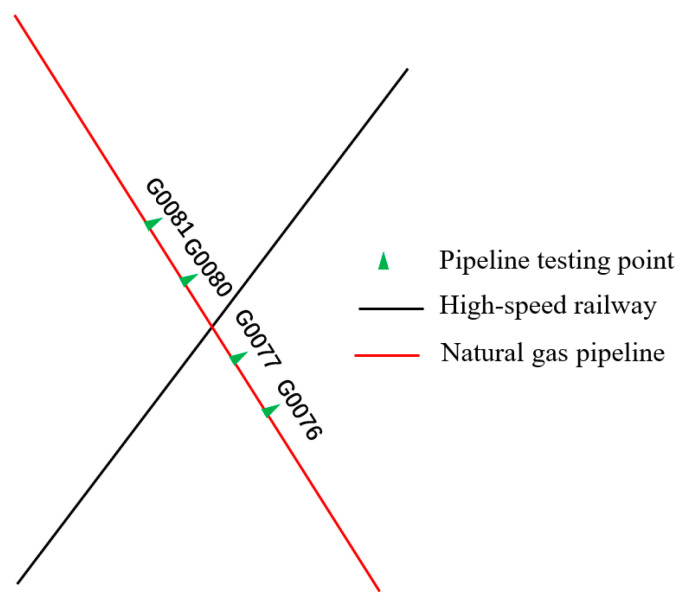
Relative positioning of Pipeline 1 and the high-speed railway line.

**Figure 4:**
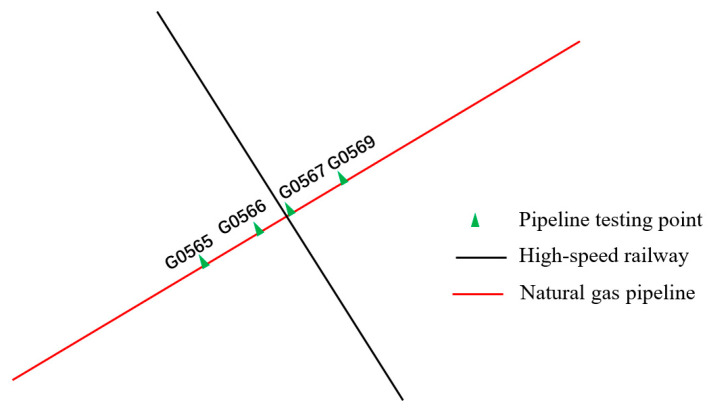
Relative positioning of Pipeline 2 and the high-speed railway line.

**Figure 5:**
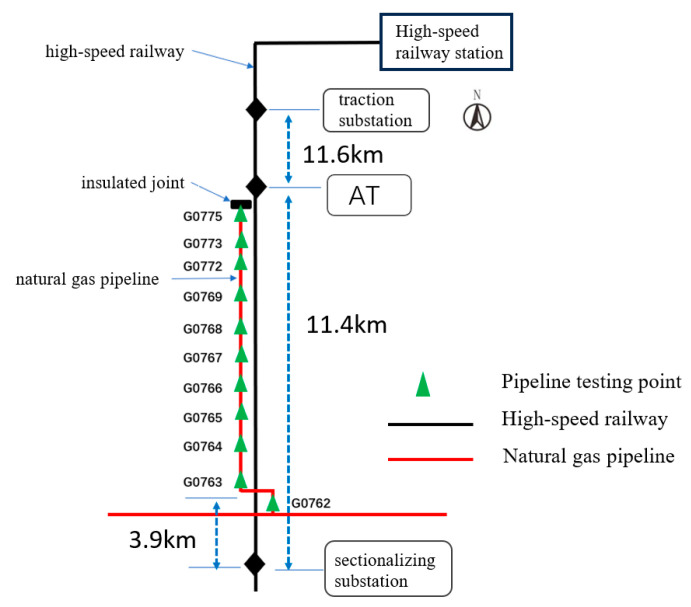
Relative positioning of Pipeline 3 and the high-speed railway line.

This study focuses on three pipelines. Pipeline 1 and Pipeline 2 intersect with the high-speed railway line without running parallel (refer to Figure 3 and Figure 4), while Pipeline 3 intersects and runs parallel to the high-speed railway line (refer to Figure 5). On the north side of Pipeline 3, there is an insulated joint located at the G0775 test pile position. It starts running parallel to the high-speed railway line for approximately 7.5 km. Pipeline 3 intersects the railway line at two points, with the intersection point situated on the south side of Pipeline 3. Additionally, there is a longer extension of Pipeline 3 in the east–west direction on its south side. The specific relative positioning of Pipeline 3 can be observed in Figure 5. All three pipelines are made of X60 material and have a 3PE anti-corrosion coating. Cathodic protection is employed for the pipelines. The high-speed railway line adopts the AT power supply method. At the starting point where Pipeline 3 begins running parallel, there is an AT power supply station. Located 11.6 km to the north of the AT power supply station is the traction substation, and 11.4 km to the south is the sectionalizing substation.

## 5. Test Results and Analysis

### 5.1. Test Results of Pipeline and High-Speed Railway Intersections

**Figure 6:**
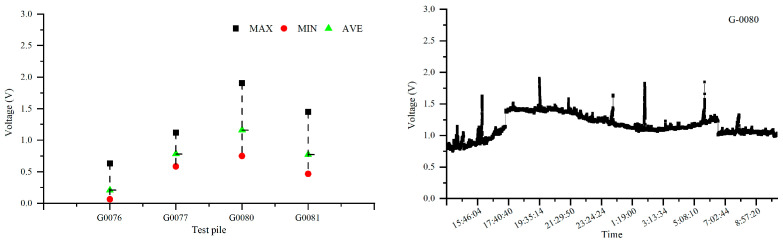
Distribution of AC voltage and 24 h AC voltage fluctuations influenced by the high-speed railway line on Pipeline 1 and test pile G0080.

**Figure 7:**
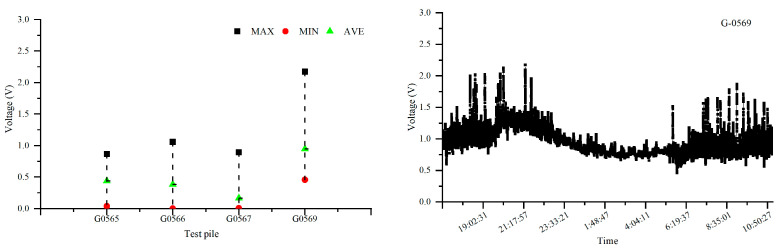
Distribution of AC voltage and 24 h AC voltage fluctuations influenced by the high-speed railway line on Pipeline 2 and test pile G0569.

Figure 6 and Figure 7 depict the 24 h distribution of alternating current voltage at test piles adjacent to the intersection of Pipeline 1, Pipeline 2, and the high-speed railway, as well as the 24 h fluctuation of alternating current voltage at the G0080 test pile location. The intersection point between Pipeline 1 and the high-speed railway line is located between test piles G0077 and G0080, while the intersection point between Pipeline 2 and the high-speed railway line is between test piles G0567 and G0569. From the figures, it can be observed that the interference caused by the high-speed railway line on Pipeline 1 results in an AC voltage of approximately 1.1 V, and for Pipeline 2, the interference causes an AC voltage of around 1.2 V. This indicates that when pipelines intersect with the high-speed railway line, they experience some level of AC interference, although the overall degree of interference is relatively small.

### 5.2. Test Results of Parallel Intersection Between Pipeline and High-Speed Railway

**Figure 8:**
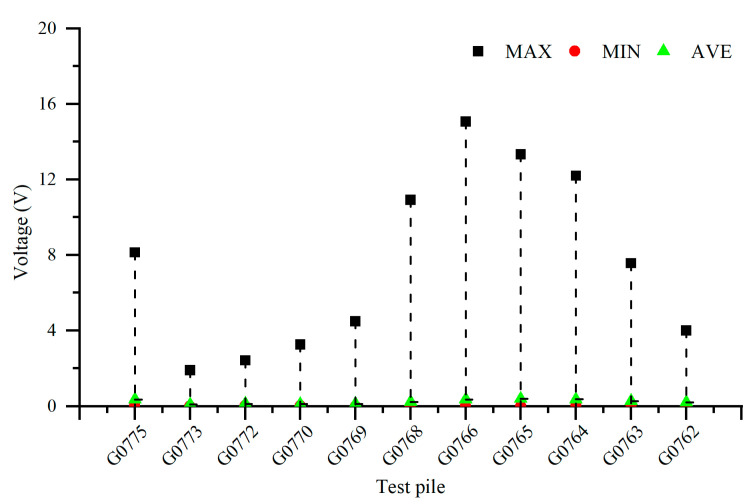
Distribution of AC voltage influenced by the high-speed railway line.

Figure 8 illustrates the 24 h distribution of AC voltage on Pipeline 3 when influenced by the high-speed railway line. From the figure, it can be observed that the interference caused by the high-speed railway line results in an AC voltage distribution ranging from 1.90 V to 15.08 V on the pipeline. Pipeline 3 experiences significant interference from the high-speed railway line, and the degree of interference varies at different test pile locations. The peak AC voltage along the pipeline occurs at test piles G0775 (insulation joint position) and G0766. When the gas pipeline intersects with the high-speed railway at certain points and runs parallel in other sections, the distribution pattern of AC voltage interference exhibits a peak at the starting point of the insulation joint, and the AC voltage decreases and then increases as it moves away from the insulation joint. The test results indicate that the interference caused by the parallel intersection between the high-speed railway line and the pipeline is much greater than that caused by a simple crossing.

### 5.3. Analysis of AC Interference Patterns on Pipelines Affected by High-Speed Railway

**Figure 9:**
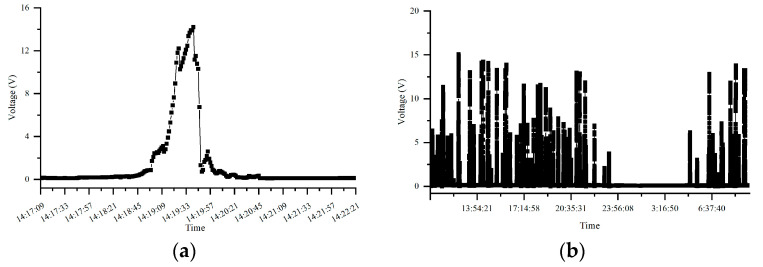
AC voltage fluctuations at test pile G0766 of Pipeline 3: (**a**): 24 h AC voltage fluctuations; (**b**): AC voltage fluctuations during a specific time period.

Figure 9 illustrates the 24 h AC voltage fluctuations and a magnified view of the AC voltage fluctuations at a specific time period for test pile G0766 of Pipeline 3 when influenced by the high-speed railway line. From the figure, it can be observed that when the natural gas pipeline is affected by the high-speed railway line, the AC voltage exhibits pulsating fluctuations. During the time periods when trains pass through, the AC voltage gradually increases and then decreases again. When no trains are passing through, the pipeline experiences no AC voltage and remains free from interference.

**Figure 10:**
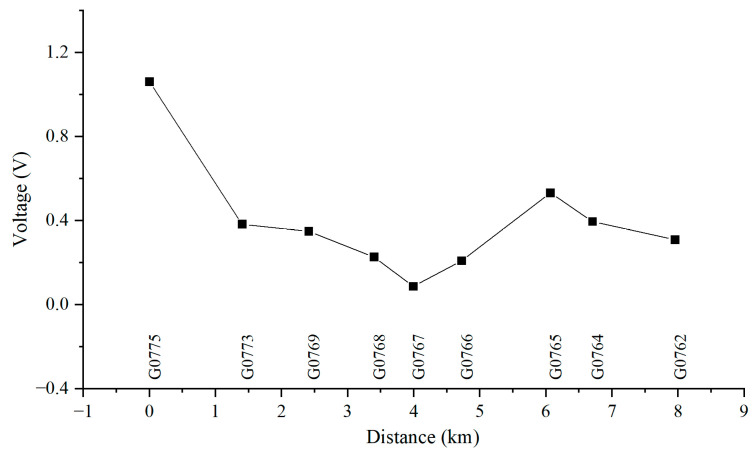
Distribution of AC voltage when the train runs near the G0775 test pile.

**Figure 11:**
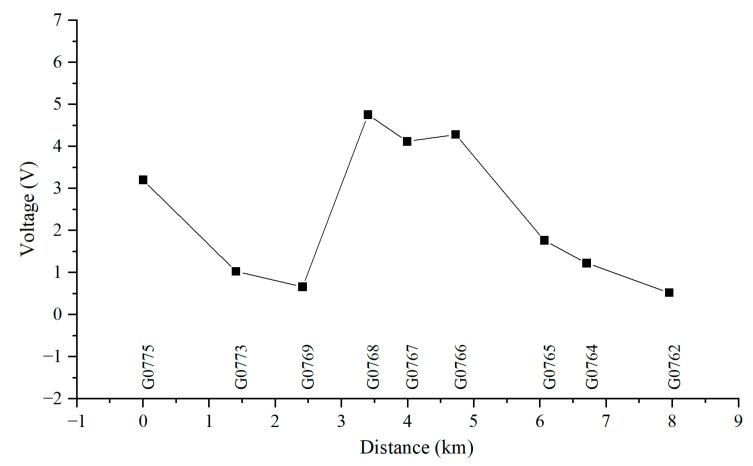
Distribution of AC voltage when the train runs near the G0768 test pile.

**Figure 12:**
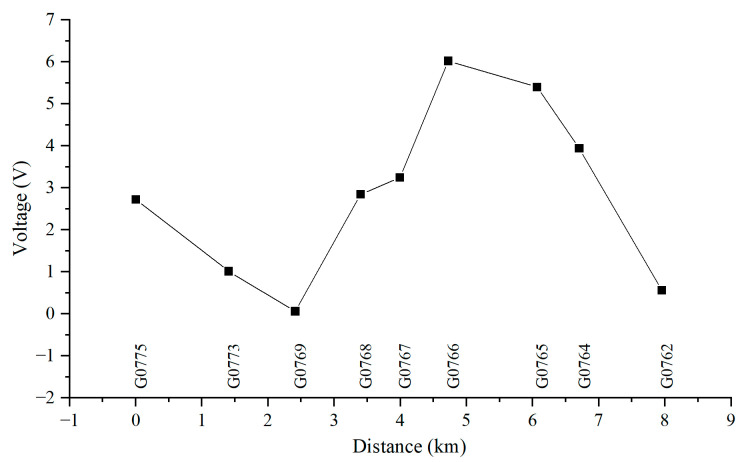
Distribution of AC voltage when the train runs near the G0766 test pile.

**Figure 13:**
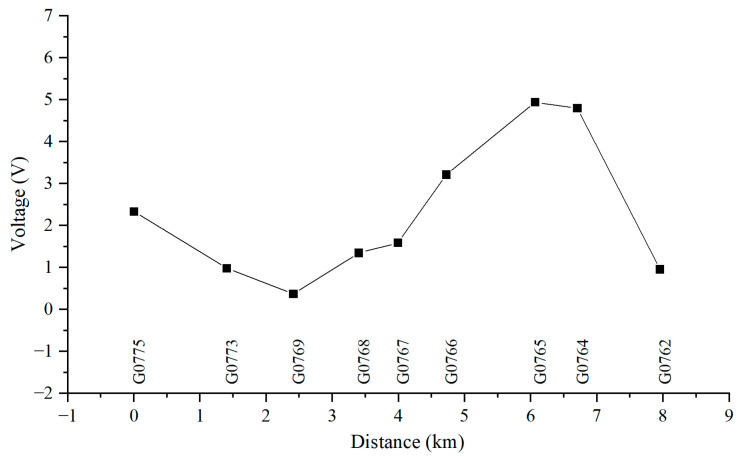
Distribution of AC voltage when the train runs near the G0764 test pile.

Figure 10, Figure 11, Figure 12 and Figure 13 show the distribution of AC voltage along the pipeline when the high-speed railway train runs near the G0775, G0768, G0766, and G0764 test piles, respectively. As shown in Figure 10, when the train runs near the G0775 test pile, the maximum AC voltage occurs at the G0775 test pile. The distribution of AC voltage along the pipeline decreases first and then increases as it moves away from the G0775 test pile, and another AC voltage peak appears at the G0765 test pile. Figure 11, Figure 12 and Figure 13 reveal that when the train runs near the G0768, G0766, and G0764 test piles, the corresponding locations of the pipeline have peak AC voltage values. As the distance from the test piles where the train is located increases, the AC current gradually decreases. Another AC voltage peak appears at the G0775 test pile location. It can be observed that regardless of the train’s position, the G0775 test pile location consistently exhibits a peak point of AC voltage distribution. Nevertheless, in all cases, an additional AC voltage peak consistently appears at the G0775 test pile. Since G0775 is located at the pipeline insulation joint and near the AT substation of the high-speed railway, this location is identified as a characteristic point where significant AC voltage interference occurs, mainly due to potential accumulation rather than current intensity.

**Figure 14:**
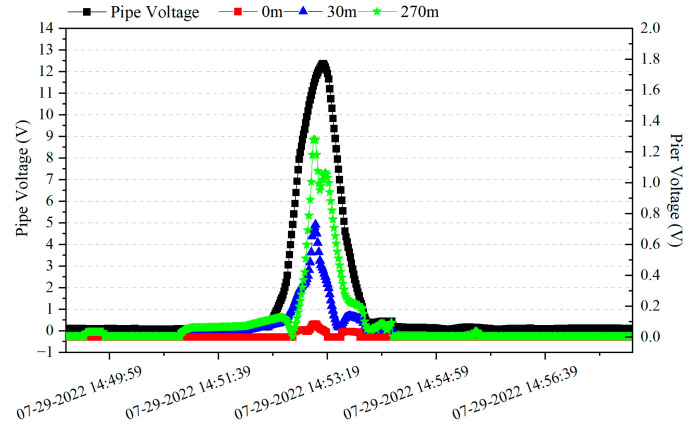
Distribution of AC voltage and time between the G0766 test pile and the high-speed railway line pier.

Figure 14 shows the distribution of alternating current voltage at the G0766 test pile and the high-speed railway track support-to-ground voltage when a train passes, as monitored synchronously. The AC voltage between the high-speed railway line pier and the ground was tested with reference electrodes placed near the pier, at a position 30 m away from the pier, and at a position 270 m away from the pier. The high-speed train passed the G0766 test pile at 14:53:07, and the peak value of AC voltage between the high-speed railway line pier and the ground occurred at 14:53:08, while the peak value of AC voltage at the G0766 test pile occurred at 14:53:14. From Figure 14, it can be observed that the fluctuation pattern of the pipeline’s AC voltage is similar to that of the AC voltage at the high-speed railway line pier, but there are differences in the timing of peak values. When the train passes, the alternating current at the high-speed railway line pier increases significantly, indicating that the support leaks alternating current when the train passes, and the moment when the train passes reaches the maximum leaked alternating current. The fluctuation pattern of the pipeline’s AC voltage is close to that of the high-speed railway line pier, indicating that the AC interference on the pipeline is partially caused by resistive coupling interference from the leaked AC current at the pier. However, there is a noticeable time difference between the peak points of AC voltage on the pipeline and the peak points of AC voltage at the pier, indicating that the pipeline is also affected by non-resistive coupling interference.

**Figure 15:**
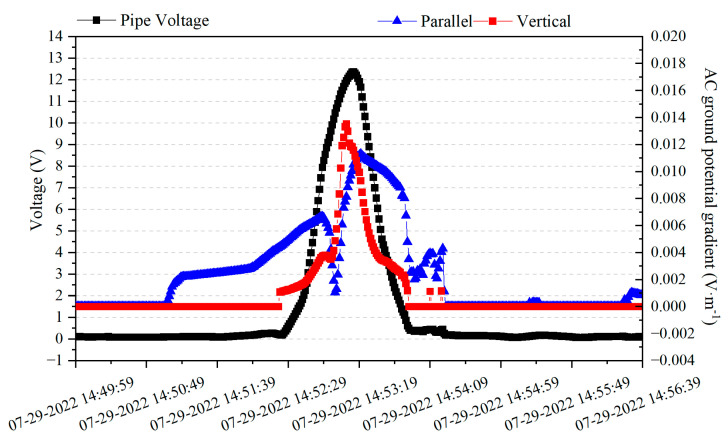
AC voltage and AC ground voltage gradient–time distribution of the G0766 test pile.

Figure 15 shows the AC voltage–time distribution and AC ground voltage gradient–time distribution of the synchronized monitoring of the G0766 test pile during the passage of the train. The AC ground voltage gradient monitoring point is selected near the G0766 test pile, parallel and perpendicular to the high-speed rail line. From Figure 15, it can be observed that the peak value of the AC ground voltage gradient perpendicular to the high-speed rail line occurs at 14:53:09, which is close to the peak value of the AC ground voltage of the high-speed rail pier to the ground. Both occur at the time when the train passes, indicating that the peak value of the resistive coupling interference caused by the train passage is reached.

The AC ground voltage gradient parallel to the high-speed rail line starts to rise at a distance of 10.25 km from the test point (14:51:04). It starts to decrease at a position 1.16 km away from the test point (14:52:53). It reaches the lowest value at a distance of approximately 0.25 km from the high-speed rail passing by (14:53:03) and then starts to rise again. It reaches its peak value at a position approximately 1.17 km away from the test point after the high-speed rail passes by (14:53:20) and then starts to decrease. This indicates that the peak value of the electromagnetic coupling interference caused by the high-speed rail on the pipeline is not at the location where the train is traveling.

The peak time for the resistive coupling interference caused by the high-speed rail is 14:53:08, and the peak time for the electromagnetic coupling interference caused by the high-speed rail is 14:53:20, and the peak point of the AC interference on the pipeline occurs at 14:53:14, with a certain time difference from the peak points of the resistive coupling interference and the electromagnetic coupling interference. This indicates that the AC interference on the pipeline is mainly caused by the superposition of these two types of interference and appears at an intermediate position between the two time points.

## 6. Analysis of Interference Patterns Based on Numerical Simulation

In order to clarify the principle of high-speed railway interference on pipelines, this paper uses numerical simulation software CDEGS 15.4 to establish an AC interference model of high-speed railway on Pipeline 3, simulating the AC interference of the high-speed railway on the pipeline. Pipeline 3 is made of X60 material, with a diameter of 508.0 mm, buried at a depth of 1.5 m, and a wall thickness of 10.0 mm. The pipeline’s anti-corrosion layer is 3PE, and the pipeline adopts external current protection.

The high-speed railway adopts the auto-transformer (AT) power supply mode. In this mode, an auto-transformer is connected between the catenary wire (CW) and the feeder line (AF). In this study, the traction substation of the high-speed railway is modeled as a simulated AC voltage source of 27.5 kV at 50 Hz. This frequency corresponds to the actual traction power used in high-speed railways.

Due to the action of the auto-transformer, the return current on the steel rail flows back to the substation through the winding of the auto-transformer and the feeder line. During normal operation, the high-speed railway is unilaterally powered, and the power supply systems in different sections are mutually isolated. Different power supply sections generally do not cross-supply, and the power supply system supplies power to the locomotives through the traction substation, auto-transformer, electric locomotive, rail, and feeder line. Auto-transformers with a turns ratio of 2:1 are included at the traction substations and at two AT locations, totaling three positions. At the position parallel to the start of Pipeline 3 (G0775 test pile position) is one AT substation, with the traction substation located 11.6 km north of the AT substation and the sectionalizing substation located 11.4 km south of the AT substation. Grounding grids are drawn at the traction substation and 2 AT locations, directly connected to the rails, with a size of 66 m × 66 m. The electrical parameters of the high-speed railway conductors are shown in Table 1. The train is replaced by an equivalent impedance, with an equivalent resistance of 45 Ω (the operating current of the train is approximately 600 A). The electrical parameters of conductors of the high-speed railway are shown in Table 1.

A homogeneous soil model with a soil resistivity of 55 Ω·m is used for this calculation.

**Figure 16:**
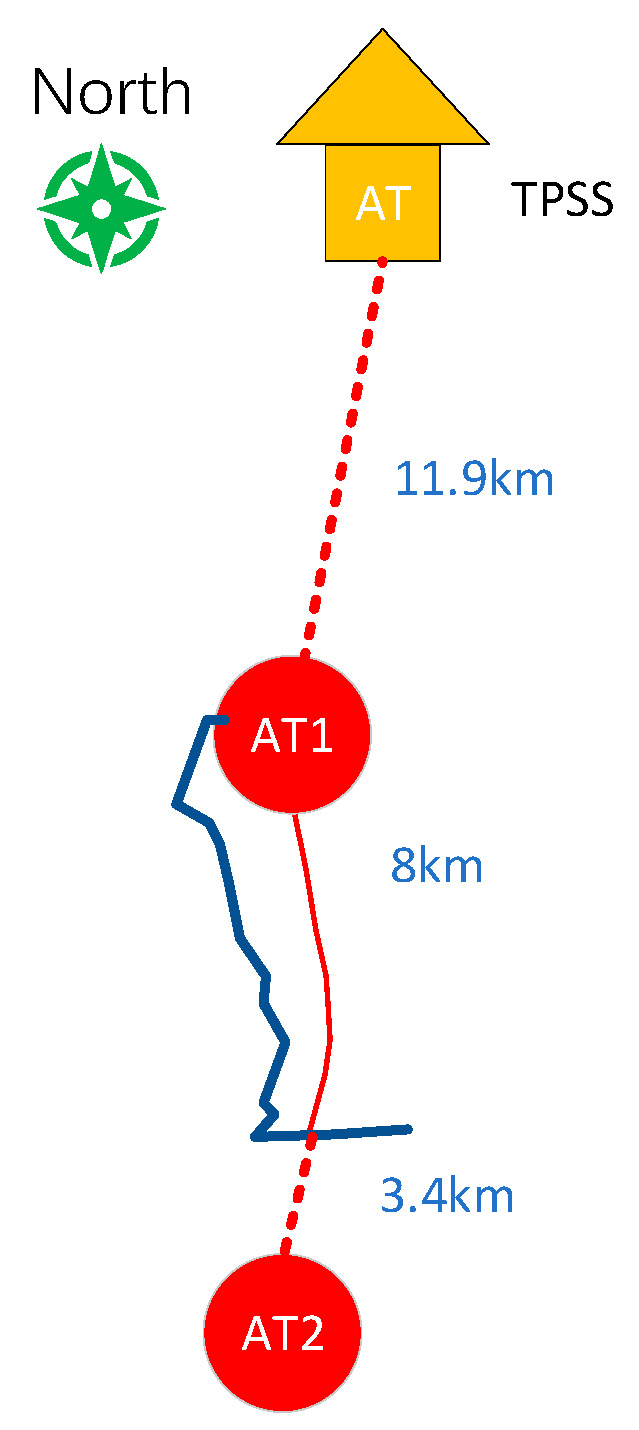
Model of AC interference calculation for pipelines.

Based on the cross-parallel relationship between the pipeline and the high-speed railway described above, as well as the relevant data, the model is established as shown in Figure 16.

To verify the accuracy of the calculation model, field tests were conducted to measure the interference on the G0762–G0775 test piles on Pipeline 3 when the train passes near the G0766 test pile. The test results were compared with the calculated results to validate the accuracy of the calculations.

**Figure 17:**
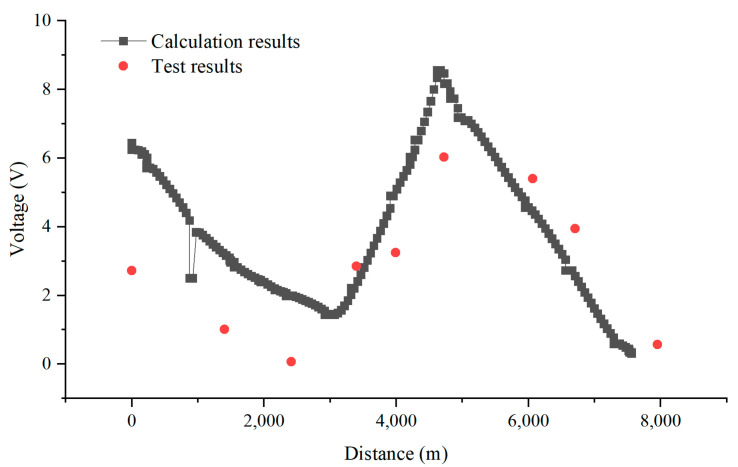
Comparison between calculation results and test results.

The model was calibrated using the synchronized test results along the entire pipeline, as shown in Figure 17. As seen in the figure, the field test results align with the trend of the calculated results. When the train reaches near the G0766 test pile, the corresponding position on the pipeline experiences peak AC voltage, which gradually decreases as the distance from the test pile location increases. This indicates that the model can effectively represent the actual pipeline conditions.

**Figure 18:**
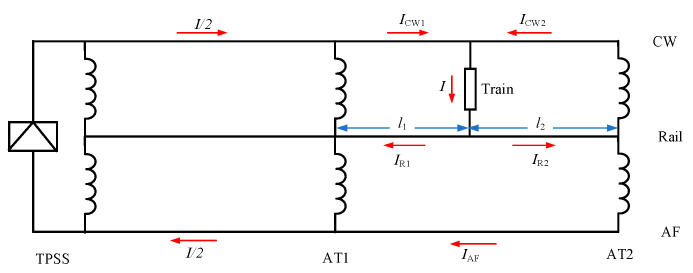
Current distribution diagram of the train in the AT1 and AT2 sections.

**Figure 19:**
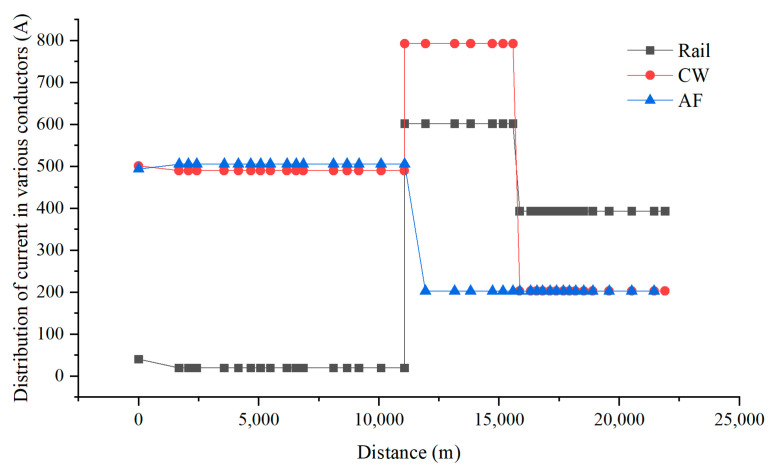
Distribution of current in various conductors of the high-speed railway.

When the train is running between AT1 and AT2, the current distribution in each conductor under ideal conditions is shown in Figure 18. According to Kirchhoff’s laws, neglecting leakage impedance and assuming that the longitudinal impedance of the conductors is directly proportional to their length, we can obtain Equations (1)–(5). In order to analyze the reason why the AC voltage at the corresponding position of the pipeline near the G0766 test pile peaks when the train passes by, the current distribution in the catenary wire (CW), feeder line (AF), and rail (Rail) was calculated, as shown in Figure 19.

(1) $$I_{\mathrm{CW}1}+I_{\mathrm{CW}2}=I$$

(2) $$I_{R1}+I_{R2}=I$$

(3) $$I_{R2}=I_{\mathrm{CW}2}+I_{\mathrm{AF}}$$

(4) $$I_{R1}=\frac{l_{2}}{l_{1}+l_{2}}I$$

(5) $$I_{R2}=\frac{l_{1}}{l_{1}+l_{2}}I$$

From Figure 18, it can be observed that when the train reaches near the G0766 test pile, according to Equations (1)–(5), theoretically, the sum of I_CW1_ and I_R1_, I_AF_ should be equal, and the sum of I_R2_ and I_CW2_, I_AF_ should also be equal. The overall electromagnetic field generated by them depends on the imbalance in the positions of CW, AF, and Rail, as well as the current imbalance caused by leakage in Rail. From Figure 19, it can be seen that the current directions on the rail are opposite in front and behind the train. Therefore, the imbalance in current direction before and after the train is also opposite, resulting in opposite external magnetic field directions and the generation of opposing longitudinal electromotive forces. This leads to the occurrence of interference peaks at the location of the train.

In the model, the calculation of current distribution in each conductor is shown in Figure 19. It can be seen from the diagram that the current in the rail varies significantly because the rail releases current into the ground. For example, on the left side of the train, I_CW1_ is 12 A higher than the sum of I_R1_ and I_AF_, while on the right side of the train, the sum of I_CW1_ and I_AF_ is 11 A higher than I_R2_. Therefore, under the condition of induction interference, the presence of peak values at the location of the train is caused by the different directions of the synthesized currents on both sides of the train.

**Figure 20:**
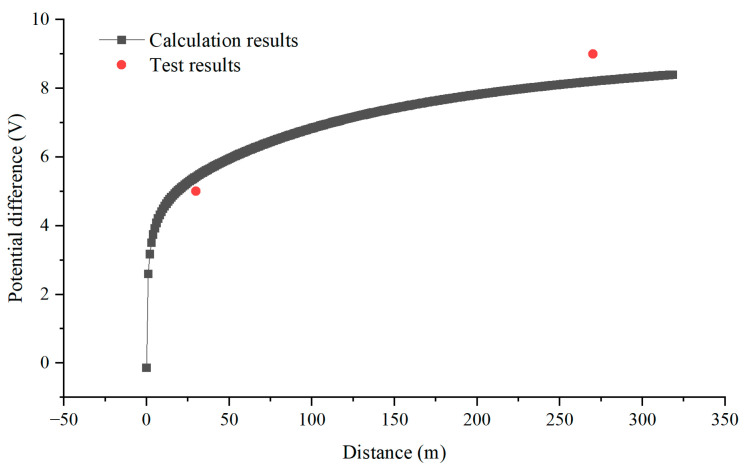
Comparison between potential difference calculation and test results.

Figure 20 illustrates the calculation of current and potential distribution in the vicinity of the nearest pier to the G0766 test pile along the pipeline. The calculation results show that the current entering the ground near the pier close to pipeline G0766 test pile is 4.98 A. At this point, the voltage drops to 5.42 V at a distance of 30 m from the high-speed railway track pier, and the voltage drops to 8.20 V at a distance of 270 m from the high-speed railway track pier. These results are consistent with the actual tested AC voltage measurements at the 30 m and 270 m positions from the pier shown in Figure 12.

**Figure 21:**
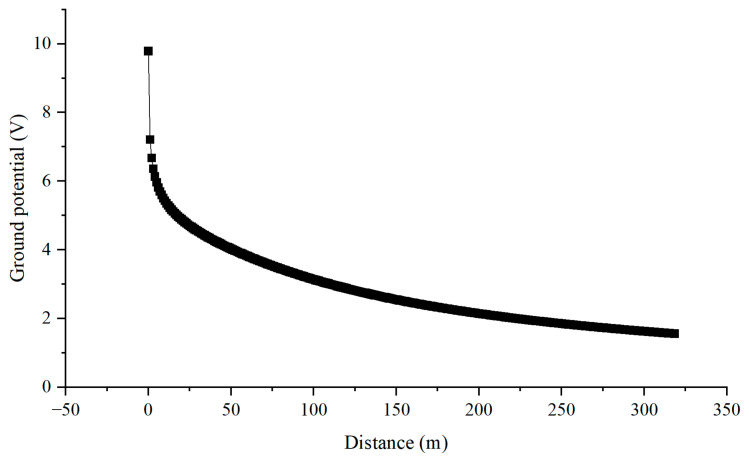
Ground potential distribution near the tower.

At a distance of 30 m from the high-speed railway track pier, the ground potential drops to 46.3% of the peak value, and at a distance of 270 m from the high-speed railway track pier, the ground potential drops to 17.9% of the peak value. From Figure 21, it can be seen that the potential of the pier to the infinite far ground is not high, and at most, it can only generate interference of around 10 V.

**Figure 22:**
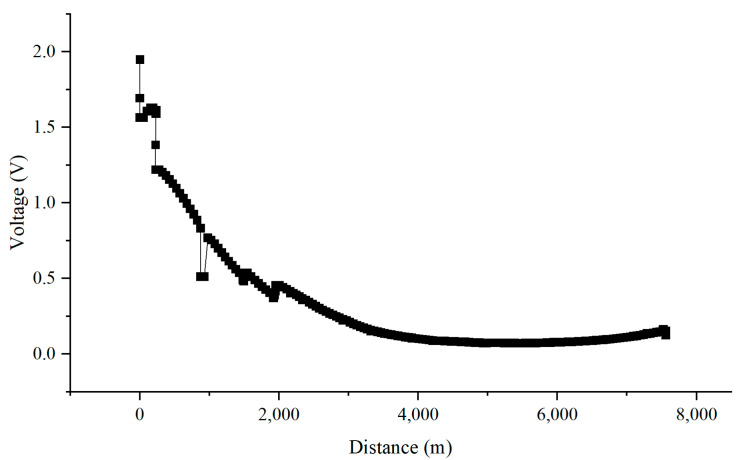
Interference voltage distribution in the presence of resistive interference.

In order to determine whether the interference suffered by the pipeline comes from resistive interference or inductive interference, the current in the calculation was changed to direct current, which means that the pipeline only suffers resistive interference. Figure 22 showed that the maximum interference voltage of the pipeline under DC conditions was 1.95 V, and the maximum interference voltage gradually decreased. However, under normal conditions, the simulation results showed that the maximum interference voltage of the pipeline could reach 8.56 V and reached a peak at the location where the train passed. Therefore, the interference caused by high-speed railway to the pipeline is mainly due to inductive coupling. Inductive coupling is caused by the interaction between the changing magnetic field generated by the high-speed train during operation and the induced current in the pipeline itself, which acts as a conductive loop. This kind of interference will lead to a higher potential difference in the pipeline.

## 7. Conclusions

In this study, field measurements and numerical simulations were carried out to investigate the AC interference between high-speed railways and nearby gas pipelines. The monitoring included pipeline AC voltage, ground potential gradients, pier-to-ground voltage, and the timing of train passages. A numerical model was also developed to explain the underlying interference mechanisms.

The main findings can be summarized as follows:

(1) The interference caused by the parallel and crossover conditions of high-speed railway and pipelines is much greater than that caused by the crossing condition alone.
(2) When the gas pipeline intersects with the high-speed railway at certain points and runs parallel in other sections, the peak point of the AC interference voltage of the pipeline will occur at the insulation joint position of the pipeline, the position of the pipeline corresponding to the high-speed railway AT, and the position of the pipeline corresponding to the train running point.
(3) The peak value of resistive coupling interference caused by high-speed railway to pipelines appears at the moment when the train passes, while the peak value of electromagnetic coupling interference caused by high-speed railway to pipelines does not appear at the moment when the train passes.
(4) The AC interference caused by high-speed railway to pipelines is caused by the superposition of resistive coupling interference and electromagnetic coupling interference. For this case, the interference caused by inductive coupling plays a dominant role.

Future research should focus on incorporating uncertainty analysis into the evaluation of interference effects, extending the study to pipelines under different soil and geological conditions, and improving the numerical model to enhance predictive accuracy and applicability to international cases.

## Figures and Tables

**Table 1:** The parameters of the high-speed railway.

No.	Data Name	Value
1	Internal resistance of CW	0.183 Ω/km
2	Internal resistance of AF	0.096 Ω/km
3	Internal resistance of Rail	0.45 Ω/km
4	Leakage resistance of Rail	15 Ω·km

## Data Availability

The original contributions presented in this study are included in the article. Further inquiries can be directed to the corresponding author.

## References

[1] Guo Y.B., Cheng L., Wang D.G., Liu S.H. (2015). Effects of alternating current interference on corrosion of X60 pipeline steel. Petrol. Sci..

[2] Qin Q.Y., Xu J., Wei B.X., Fu Q., Gao L.Q., Yu C.K., Sun C., Wang Z. (2021). Synergistic effect of alternating current and sulfate-reducing bacteria on corrosion behavior of X80 steel in coastal saline soil. Bioelectrochemistry.

[3] Guo Q.R., He W.Z., Zuo G., Dong Z.Z., Chen J.H., Li Z.P., Leng X.Y., Li Z.H. (2005). Study on the interference of electrified railway on oil pipeline and its protection method. Petrol. Eng. Constr..

[4] Wang K. (2013). Influence of Electrified Railway on Oil and Gas Pipeline and Protective Measures. Ph.D. Thesis.

[5] Xian J., Wang W.B., Mei P., Zhang H.W., Xu J., Sun C., Yan M.C., Wu T.Q., Yu C.K. (2013). Effect of Stray Current Induced by Electric Railway on Buried Pipeline. Total Corros. Control.

[6] Li N.X. (2015). Protection measures against electromagnetic interference from oil and gas pipelines by high-speed electrified railways. Guide Bus..

[7] Wei X. (2012). Study on Corrosion Interference of AC Electrified Railway on Buried Pipeline. Ph.D. Thesis.

[8] Chen L. (2015). Detection and protection measures of AC stray current interference of oil pipeline in southern Jiangsu Province. Corros. Prot..

[9] Wu C.F., Wang B., Pei Q., Zhou B.K., Chen H.Y., Xue Z.Y. (2014). AC Stray Current Testing and Evaluation of the Tieling-Qinhuangdao Pipeline. Pipeline Technol. Equip..

[10] Fickert L., Schmautzer E., Braunstein R., Lindinger M. (2010). Reduction of the electrical potential of interfered pipelines due to currents of high voltage power lines and electric railways. Elektrotech. Inftech..

[11] Linhardt P., Ball G. AC Corrosion: Results from Laboratory Investigations and from a Failure Analysis. Proceedings of the Corrosion 2006 Conference.

[12] Bosko M., Bozidar F.G., Tomislav R. (2014). Electromagnetic fields and induced voltages on underground pipeline in the vicinity of AC traction system. J. Energy Power Eng..

[13] Hosokawa Y., Kajiyama F., Nakamura Y. (2004). New cathodic protection criteria based on direct and alternating current densities measured using coupons and their application to modern steel pipelines. Corrosion.

[14] Zhu J.G. (2018). Research on Electromagnetic Interference Characteristics of Underground Oil and Gas Pipelines Caused by Electrified Railway Communication. Ph.D. Thesis.

[15] Charalambous C.A., Demetriou A., Lazari A., Nikolaidis A. (2018). Effects of Electromagnetic Interference on Underground Pipelines caused by the Operation of High Voltage A.C. Traction Systems: The Impact of Harmonics. IEEE Trans. Power Deliv..

[16] Zhang X.Y. (2010). Calculation and protective measures of electromagnetic interference induced by electrified railway on the oil and gas pipelines. Oil Depot Gas Stat..

[17] Li X.F., Liu S.X. (2019). The impact of electrified railways on AC interference voltage of buried natural gas pipelines. Petrochem. Technol..

[18] Mariscotti A. (2003). Distribution of the traction return current in AC and DC electric railway systems. IEEE Trans. Power Deliv..

[19] Kolar V., Hrbac R., Mlcak T. Measurement and simulation of stray currents caused by AC railway traction. Proceedings of the 16th International Scientific Conference on Electric Power Engineering (EPE).

[20] Sun P.Q. (2011). AC Interference induced by electrified railway on buried metal pipelines and its protective measures. Urban Gas.

[21] Braunstein R., Schmautzer E., Graz G.P. Comparison and discussion on potential mitigating measures regarding inductive interference of metallic pipelines. Proceedings of the Electrical Systems for Aircraft, Railway and Ship Propulsion (ESARS/2010).

[22] Milesevic B., Filipovic-Grcic B., Uglesic I., Jurisic B. (2018). Estimation of current distribution in the electric railway system in the EMTP-RV. Electr. Power Syst. Res..

[23] Farahani E.M., Su Y., Chen X., Wang H., Laughorn T.R., Onesto F., Zhou Q., Huang Q. (2024). AC corrosion of steel pipeline under cathodic protection: A state-of-the-art review. Mater. Corros..

[24] Lucca G. (2019). AC corrosion on pipelines: Influence of the surface layer soil resistivity in evaluating the current density by a probabilistic approach. Prog. Electromagn. Res..

